# Biomechanical effects of original equipment manufacturer and aftermarket abutment screws in zirconia abutment on dental implant assembly

**DOI:** 10.1038/s41598-020-75469-9

**Published:** 2020-10-27

**Authors:** Yu-Ling Wu, Ming-Hsu Tsai, Hung-Shyong Chen, Yu-Tsen Chang, Tsai-Te Lin, Aaron Yu-Jen Wu

**Affiliations:** 1 Department of Dentistry, Chang Gung Memorial Hospital and College of Medicine, Chang Gung University, Kaohsiung, 833 Taiwan; 2 Mechanical Engineering, Cheng Shiu University, Kaohsiung, 833 Taiwan; 3 Center for Environmental Toxin and Emerging-Contaminant Research, Cheng Shiu University, Kaohsiung, 833 Taiwan

**Keywords:** Dental materials, Prosthetic dentistry

## Abstract

The use of aftermarket computer-aided design/computer-assisted manufacturing (CAD/CAM) prosthesis components in dental implants has become popular. This study aimed to (1) compare the accuracy of aftermarket CAD/CAM screws with that of original equipment manufacturer (OEM) abutment screws and (2) examine the biomechanical effects of different abutment screws used with zirconia abutment in an implant fixture by using three-dimensional finite element analysis (FEA). Significantly different measurements were obtained for the aftermarket CAD/CAM and OEM screws. The FEA results indicated that under the same loading condition, the maximum stress of the aftermarket CAD/CAM screws was 15.9% higher than that of the OEM screws. Moreover, the maximum stress position occurred in a wide section of the OEM screws but in the narrowest section of the aftermarket screws. The stress of the OEM zirconia abutment was 14.9% higher when using the aftermarket screws than when using the OEM screws. The effect of the manufacturing differences between aftermarket and OEM screws on the clinical effect of aftermarket screws is unpredictable. Therefore, aftermarket screws should be cautiously used clinically.

## Introduction

Replacing a missing tooth with a dental implant is a popular dental treatment. Therefore, achieving long-term success and avoiding complications in dental implantation is important. The implant assembly is divided into several parts with close interlocking links. The implant assembly includes the crown, abutment, and screw and implant fixture. The screw is a critical and fragile link that affects the success or failure of the entire implant system.

Despite the high success and survival rates of dental implants^1^, clinicians may encounter various biological and mechanical complications^2^. Biological complications include the loss of osseointegration or peri-implantitis^3^, and mechanical complications include crown fracture, abutment fracture, and loosening or fracture of abutment screws^2,4^. The fracture of the abutment screw can be a serious problem. A fixture and an abutment are connected through a screw, and the incidence of abutment screw fracture ranges between 2%^5^ and 3.9%^6^. The removal of a fractured screw is always challenging, and sometimes, the entire implant fixture must be removed if the fragment of screw remaining inside the implant prevents the implant from functioning efficiently^4,7,8^. Thus, broken screws may cause irreversible damage and affect the trust involved in the doctor–patient relationship.

With advancements in computer-aided design/computer-assisted manufacturing (CAD/CAM) techniques in dentistry, clinicians can now choose prosthetic components, including abutments and screws, from the same manufacturer, which is known as the original equipment manufacturer (OEM)^9^. Several studies have reported that CAD/CAM technology can provide results comparable to those provided by conventional techniques in terms of implant survival, prosthesis survival, and technical and biologic complications^10–13^. Aftermarket manufacturers have begun to replicate the design of prefabricated OEM prosthetic implant components and produce aftermarket CAD/CAM engineered ones, such as abutments and screws, for clinical use. Clinicians can choose alternatively engineered implant components with high efficiency and low cost. However, the biomechanical effect of aftermarket prosthetic components on the entire implant system is still unclear.

Zirconia (Zr) abutments are a restorative alternative to metal abutments^14–16^. Although Zr abutments have good aesthetic outcomes and offer several clinical advantages over metal abutments, clinicians still have doubts regarding their cracking risk, especially under the high internal stress that may be caused by the screw mechanism.

Because studies on OEM and aftermarket screw mechanisms have provided unclear results, the present study aimed to (1) compare the accuracy of aftermarket CAD/CAM screws with that of OEM abutment screws and (2) examine the biomechanical effects of different abutment screws used with Zr abutment in an implant fixture by using three-dimensional (3D) finite element analysis (FEA). To the best of our knowledge, this study is the first to compare OEM and aftermarket titanium screws in an OEM Zr abutment.

## Materials and methods

**Figure 1:**
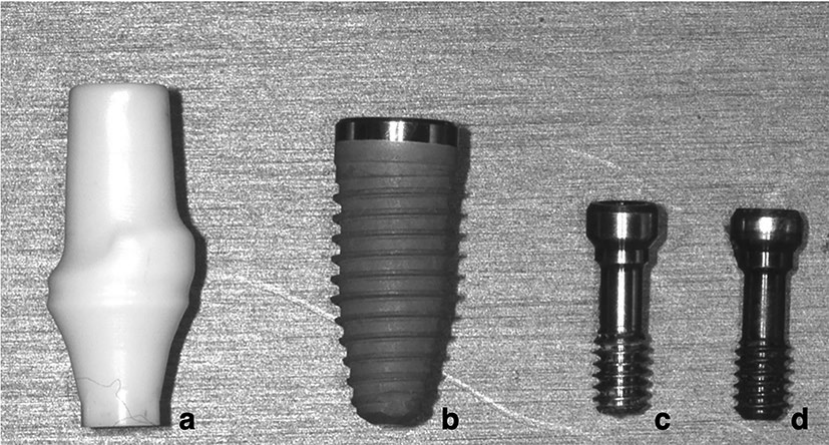
Components of the implant–abutment–screw system: (**a**) zirconia abutment, (**b**) implant fixture, (**c**) aftermarket abutment screw, and (**d**) OEM abutment screw.

A NobelReplace Conical Connection PMC dental implant (Ø = 4.3 mm × 10 mm, Nobel Biocare, Gothenburg, Sweden) and a NobelProcera zirconia abutment (Nobel Biocare, Yorba Linda, California, USA) with a full zirconia crown were used to create a morphology resembling the right central incisor. Two models were used in the analysis: (1) model I (control group) was based on the use of OEM abutment screws and (2) model II (test group) was based on the use of aftermarket CAD/CAM abutment screws. Three samples of the aftermarket abutment screws were obtained from the manufacturer (JingGang, Tainan, Taiwan), who manufactured the screws based on the data of OEM abutment screws which got from the optical scanning machine, then the manufacturer input the information to their own CAD system and made adjustment based on experience, finally milled the screw with CAM system (Fig. 1).

### Measurement of the size of the OEM and aftermarket CAD/CAM abutment screws

**Figure 2:**
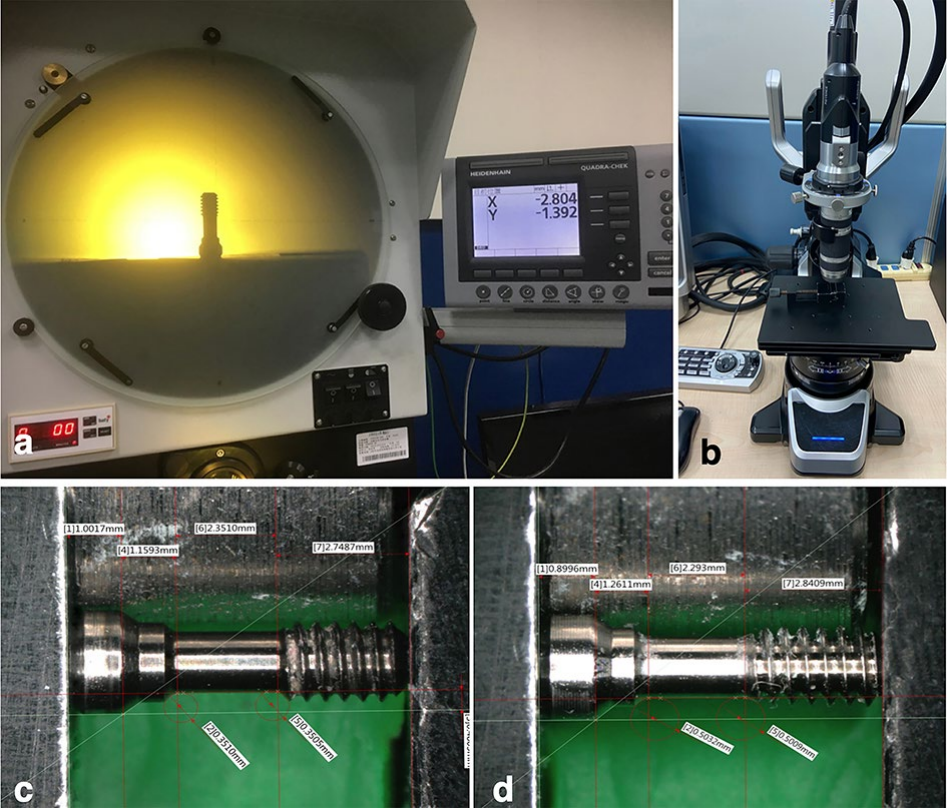
Measurement of the size of the screw samples: (**a**) scanning of the screws with a 3D optical scanning system, (**b**) use of a 3D microscope to photograph the screws with holding device and measure their size, (**c**) image of an OEM screw obtained with the 3D microscope under a magnification of 50 ×, and (**d**) image of an aftermarket screw obtained with the 3D microscope under a magnification of 50 ×.

Samples of the screws were scanned with a 3D optical scanning system (SmartScan-HE, Breuckmann, Germany; Fig. 2a), and the initial images of screws were input to the CAD using drawing software (Inventor 2017, Autodesk Inc., San Rafael, CA, USA). A holding device was then used to achieve the correct position and the parallelism of the sample screws. After ensuring accurate clamping, a 3D microscope (VHX-900F, Keyence Corporation, Osaka, Japan; Fig. 2b) was used under magnifications of 50 × to obtain photos of the screws (Fig. 2c,d). Five measurements of each sample were obtained from the photos of two models using software program (VHX-H2M2, VHX-H4M, Keyence Corporation, Osaka, Japan) by two researchers to ensure repeatability and reproducibility. The data were statistically analyzed by independent samples t-test using SPSS software (version 22.0, SPSS Inc., Chicago, IL, USA) to compare the measurements between two models. Finally, the measured dimensional data were drawn into a complete CAD system. The CAD was then imported into the ANSYS software package (ANSYS Workbench 14.5, ANSYS, Inc., USA) for analysis.

### CAD geometry modelling and mesh generation

**Figure 3:**
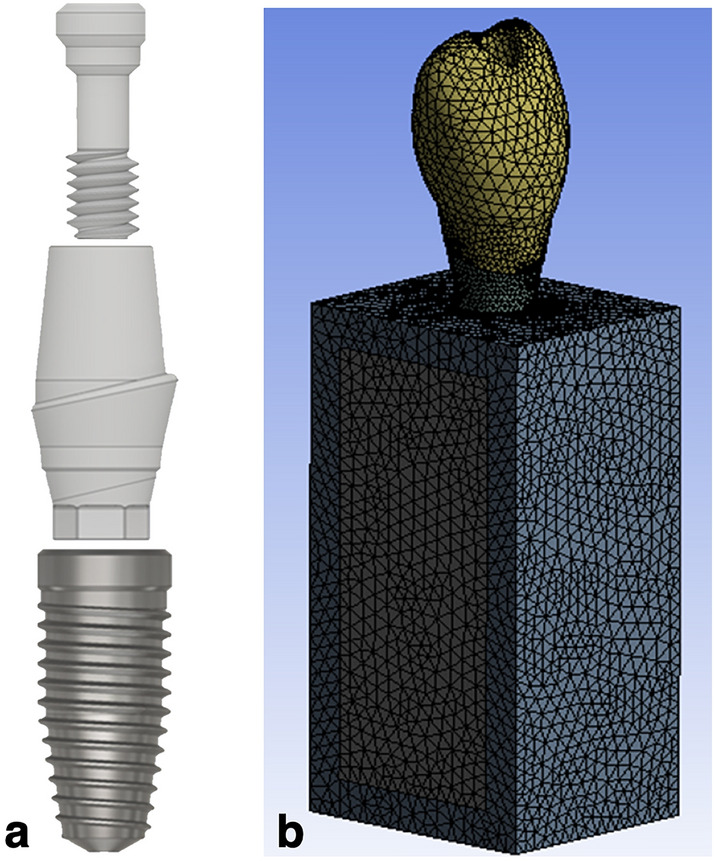
(**a**) 3D CAD models of the implant–abutment–screw complex and (**b**) a 3D finite element model of an implant and the surrounding bone.

Two CAD models were developed with CAD software (Inventor 2017, Autodesk Inc., San Rafael, CA, USA) using 3D microscope measurements and cloud data extraction (Fig. 3a). Model I (control group) consisted of the NobelReplace Conical Connection PMC implant fixture, NobelProcera zirconia abutment, and OEM abutment screw. Model II (test group) consisted of the NobelReplace Conical Connection PMC implant fixture, NobelProcera zirconia abutment, and aftermarket CAD/CAM abutment screw. The implant–abutment–screw systems were placed into a bone block model with a height of 25 mm, width of 12 mm, and thickness of 10 mm. The bone block model consisted of a spongy centre surrounded by a 2-mm cortical bone^17^. The implant fixtures were positioned in the cortical and cancellous bone blocks (Fig. 3b). All components were meshed with triangular elements, and each group comprised a similar number of elements. The number of elements in each group is presented in Table 1.

**Table 1:** Element numbers for all components of the finite element models.

	Screw	Abutment	Implant	Bone	Sum
Model I	60,413	44,764	46,482	158,332	309,991
Model II	60,739	44,611	46,797	158,313	309,991

### Boundary conditions and constraints

In this study, we assumed that the implant, abutment, and screws were homogeneous and had isotropic elastic properties^18^. The material properties of the bone and implant components (Table 2) were collected from reliable resources and published data^19–22^. To confirm the materials of the screws, abutment, and implant, energy-dispersive X-ray spectroscopy (JSM-6360, JEOL, Japan) was performed. The OEM and aftermarket CAD/CAM screws were confirmed to be made of TiAl6V4, and the dental implant was confirmed to be made of pure titanium.

**Table 2:** Properties of the materials adopted in this study.

Material	Young’s modulus (GPa)	Poisson’s ratio	References
Cortical bone	13.4	0.30	Akca et al.^19^
Cancellous bone	1.37	0.30	Akca et al.^19^
Titanium implant	115	0.35	Teixera et al.^20^
Titanium alloys (screw)	110	0.33	Pierrisnard et al.^21^
Zirconia abutment	200	0.31	Kohal et al.^22^

The implant–abutment, implant–screw, and abutment–screw interfaces were set as contacts and had a frictional coefficient of 0.3^23^. The interface between the implant and bone was also set as a contact. The frictional coefficients for the surface contacts of the rough implant surface with the cortical bone and trabecular bone were assumed to be 0.65^24^ and 0.77^25^, respectively.

### Loading conditions

For the screw tightening torque, 35 N·cm torque was applied and the axial force was created and pointing down the screw’s axis which was calculated to be 511 N with the Eq. (1) ^26^.

1 $$T \, = \, KDF$$

In Eq. (1), *T* is the tightening torque (N·m), *K* is the torque coefficient, *D* is the screw diameter (m), and *F* is the axial force (N).

**Figure 4:**
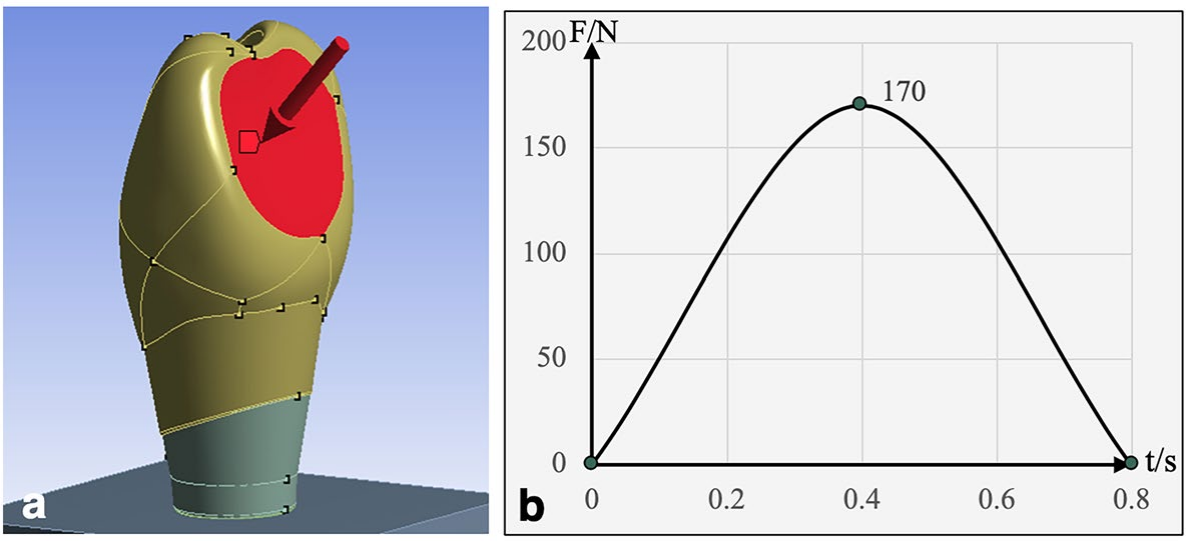
Loading direction and mode: (**a**) lateral force of 170 N applied to the palatal surface of the crown at 45° relative to the long axis of the implant and (**b**) amplitude of the loading (varying from 0 to F with a semisinusoidal pattern; the loading period was 0.8 s).

A lateral force of 170 N was applied on the entire palatal surface of the crown at 45° relative to the long axis of the implant (Fig. 4a)^27^. The loading period was 0.8 s. The amplitude of the loading varied from 0 to F in accordance with a semisinusoidal pattern (Fig. 4b)^17^.

### FEA analysis

In this study, we selected two models with the OEM and aftermarket CAD/CAM abutment screws to investigate the stress distribution in the implant–abutment–screw connection system. For a direct and systematic comparison, the same load conditions, boundary conditions, and constraints were applied for the two models. ANSYS Workbench (version 14.5, Swanson Analysis Systems) was installed on a desktop computer with a Pentium 4 processor, 16 GB of memory, and a Windows 7 operating system to analyse model data and perform stress analysis of the implant system subject to an oblique periodical loading^17^.

## Results

### Comparison of the sizes of the OEM and aftermarket CAD/CAM abutment screws

**Figure 5:**
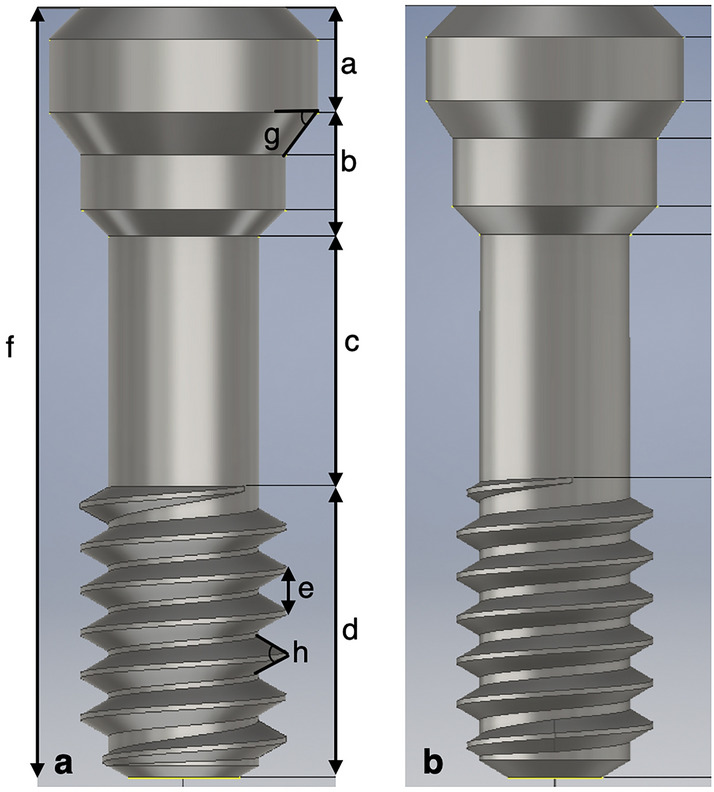
CAD geometry model and definition of the screw section: (**a**) model I, OEM screw; and (**b**) model II, aftermarket CAD/CAM screw. The letters ‘a’ to ‘f’ denote the head length, neck length, shank length, thread length, thread spacing, and overall screw length, respectively. The letters ‘g’ and ‘h’ denote the angles of the screw hex and thread, respectively.

**Figure 6:**
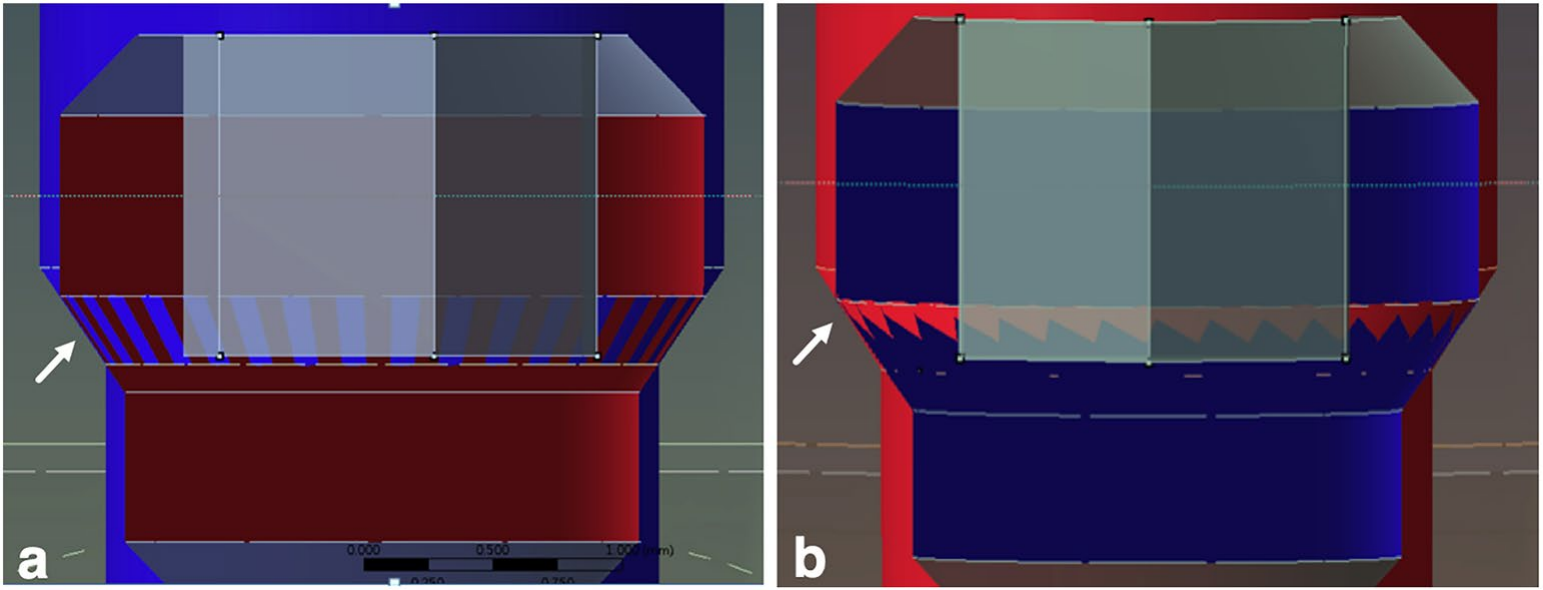
Hex angles of the two models. (**a**) Model I (OEM) has a hex angle of 55.2°, and its screw has a surface-to surface contact with the abutment. (**b**) Model II (aftermarket CAD/CAM) has a hex angle of 52.3°, and its screw has a line-to-line contact with the abutment.

A 3D microscope was used to measure the sizes of the model I (OEM) and model II (aftermarket CAD/CAM) screws. Eight sets of measurements were conducted of parameters such as the screw length (head, neck, shank, thread, and overall screw length) and screw angle (hex and thread angle) (Fig. 5). The results indicated that models I and II were statistically different in terms of eight sets of measurements (p < 0.05) (Table 3). The hex angle is defined as the retracted angle between the screw head and the neck. The differences in the hex angles of the two models caused differences in the contacts between the corresponding screws and abutments. The screw–abutment hex contact of model I was a surface-to-surface contact, whereas that of model II was a line-to-line contact (Fig. 6).

**Table 3:** Different parts of measurements of the screws.

		Model	N	Mean	SD	*p*-value
a	Head height	I	5	1.000 mm	0.001	< 0.001
		II	5	0.900 mm	0.001	
b	Neck height	I	5	1.160 mm	0.001	< 0.001
		II	5	1.261 mm	0.002	
c	Shank length	I	5	2.350 mm	0.001	< 0.001
		II	5	2.298 mm	0.003	
d	Thread length	I	5	2.749 mm	0.004	< 0.001
		II	5	2.841 mm	0.002	
e	Thread spacing	I	5	0.393 mm	0.001	0.012
		II	5	0.399 mm	0.004	
f	Overall length	I	5	7.259 mm	0.004	< 0.001
		II	5	7.301 mm	0.006	
g	Hex angle	I	5	55.223$^\circ$	0.136	< 0.001
		II	5	52.404$^\circ$	0.266	
h	Thread angle	I	5	59.036$^\circ$	0.009	0.002
		II	5	59.638$^\circ$	0.298	

Models I (OEM) and II (aftermarket CAD/CAM) exhibit statistical differences in the measurements of ‘a’ to ‘h’ (p < 0.05).

*SD* standard deviation.

### Overall stress distribution patterns and maximum von Mises stress

**Figure 7:**
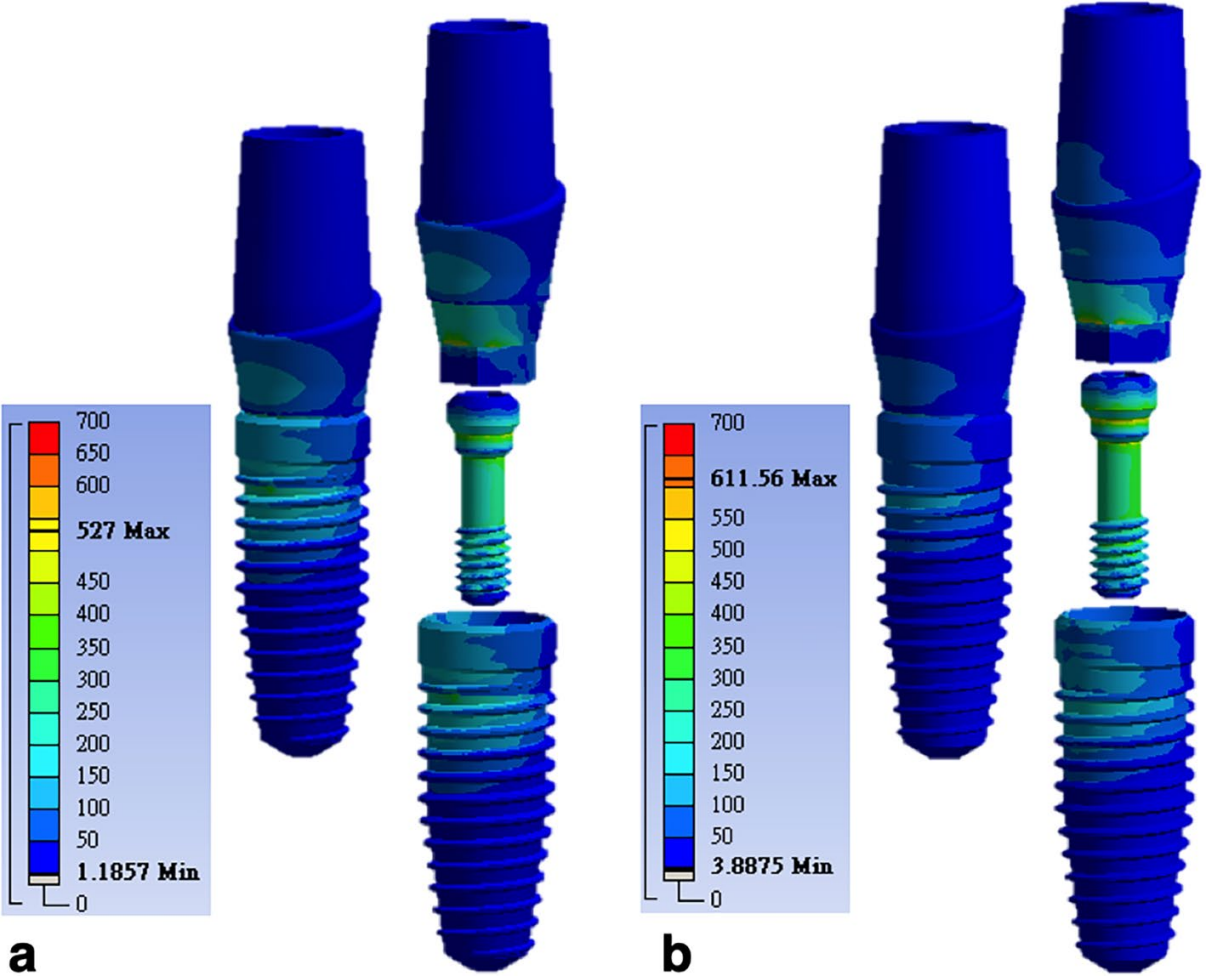
Stress distribution and maximum von Mises stress of the two models under the same loading condition: (**a**) model I (OEM) and (**b**) model II (aftermarket CAD/CAM).

**Figure 8:**
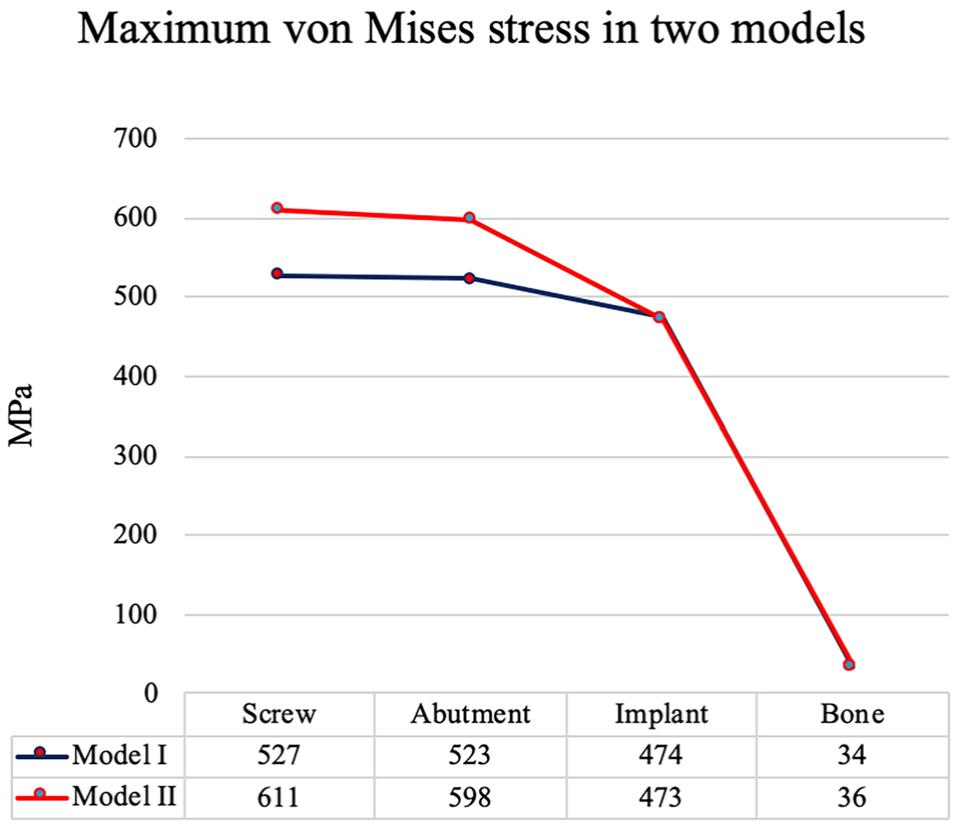
Maximum von Mises stress values for all the components of the two models. The highest level of stress was observed in the screws, and the maximum von Mises stress at the screw was 15.9% higher in model II than it was in model I.

Under the same loading conditions, the stress distributions of model I (OEM) and model II (aftermarket CAD/CAM) were similar. The stress was concentrated at the screw, abutment neck (the connection section where the abutment was inserted deep into the implant), and also the collar, first, second, and third threads of the implant (Fig. 7). The maximum von Mises stress occurred on the screws, followed by the abutments and implants. The least stress occurred in the surrounding bones (Fig. 8). The maximum stress at the screw was 527 MPa in model I and 611 MPa in model II. The maximum stress was 15.9% higher at the aftermarket screw than it was at the OEM screw. The maximum stress in the abutment was 523 MPa for model I and 598 MPa for model II. Thus, the maximum stress at the abutment was 14.3% higher in model II than it was in model I. The maximum stresses in the implant (0.2% lower in model II compared with model I) and bone (5.9% higher in model II compared with model I) were similar for both models.

### Cross-sectional maximum von Mises stress distribution patterns of screws

**Figure 9:**
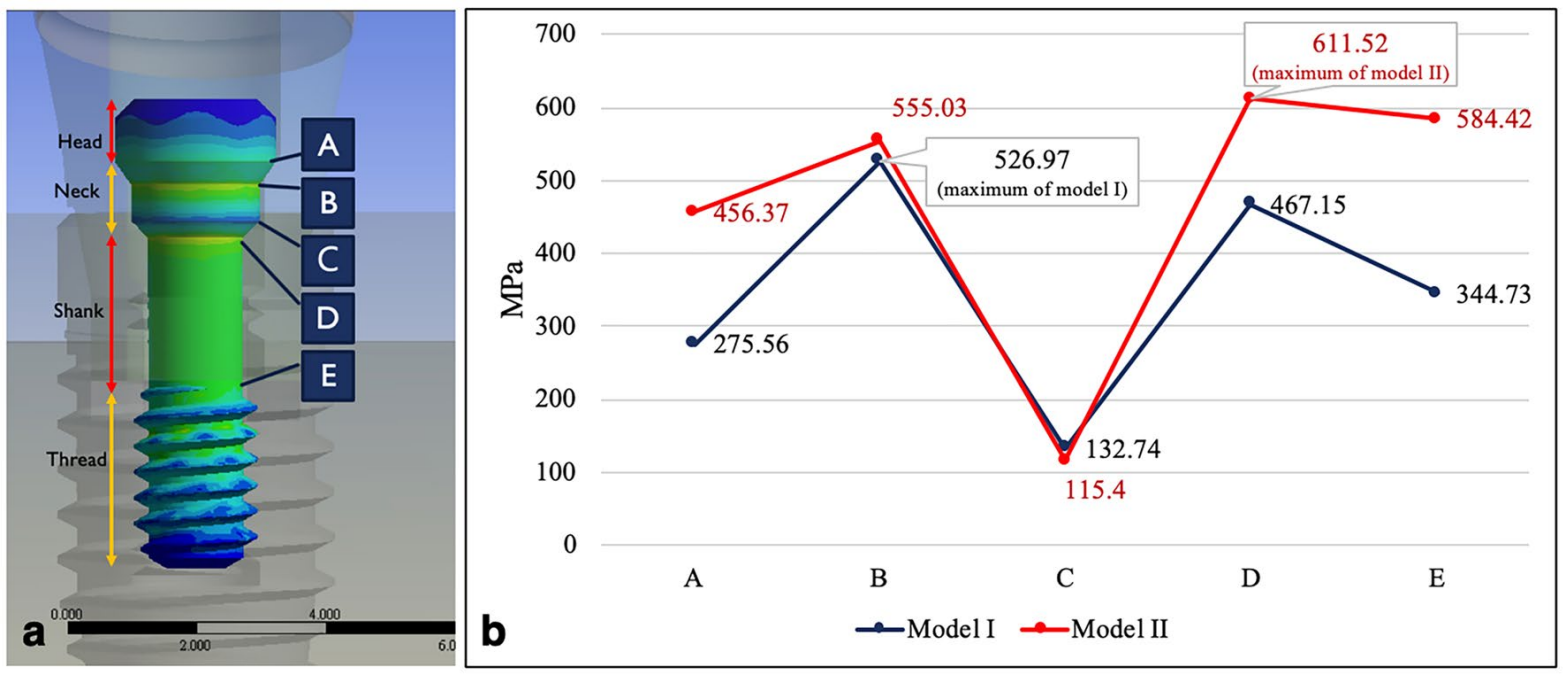
Cross-sectional maximum von Mises stresses of the screws in the two models: (**a**) analysis of the stress over section A to section E and (**b**) variation in the von Mises stresses on the screws in different sections. The overall maximum stress of the screws occurred in section B for model I (526.97 MPa) and section D for model II (611.52 MPa).

**Figure 10:**
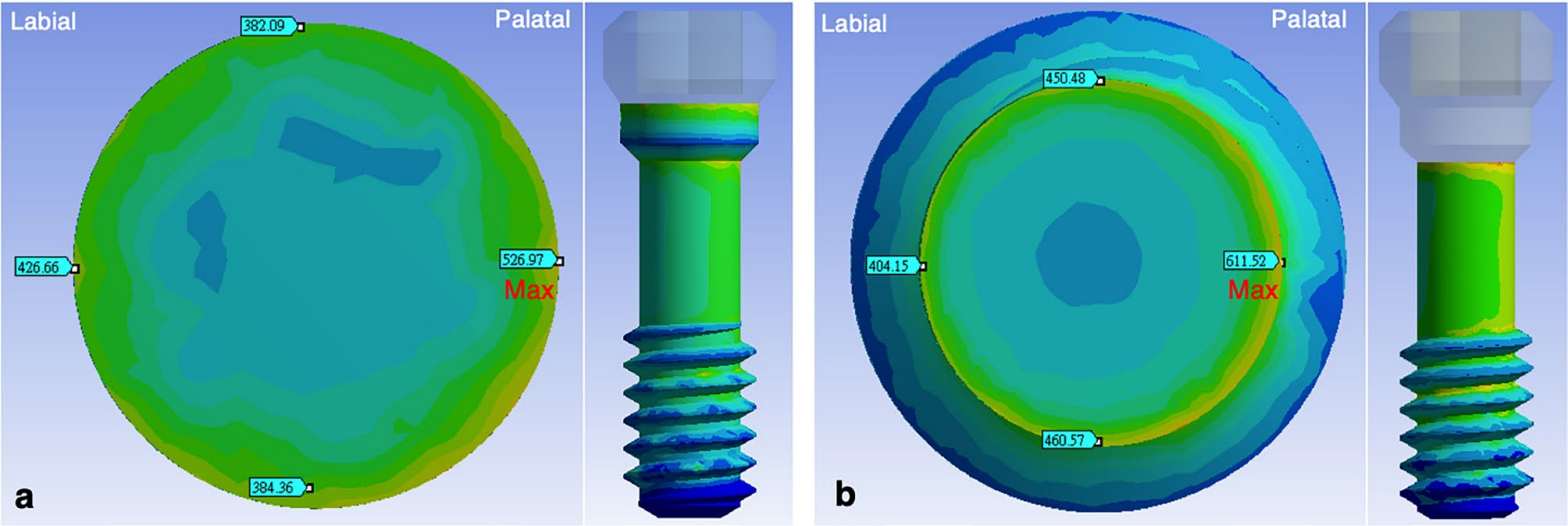
Maximum stress of each section in models I and II (the maximum stress occurred on the palatal side of both models): (**a**) model I (section B) and (**b**) model II (section D).

In the cross-sectional analysis of the screws (Fig. 9), the overall maximum stress of model I (526.97 MPa) appeared on the palatal side of section B (Fig. 10a) and that of model II (611.52 MPa) appeared on the palatal side of section D (Fig. 10b). The maximum stresses in sections A, B, D, and E were 65.6%, 5.3%, 30.1%, and 69.5% larger in model II (aftermarket CAD/CAM) than those in model I (OEM). The maximum stress in section C was 13.1% smaller in model II than that in model I.

## Discussion

We compared the sizes of the OEM and aftermarket CAD/CAM abutment screws. The results indicated that statistical differences existed in the eight measurements, including the different screw lengths and angles. The measurement error caused by the manufacturer preprocessing problem in 3D optical scanning procedure, CAD and the postprocessing error in the accuracy of CAM are possible reasons for the differences in the sizes of the CAD/CAM screws fabricated by different manufacturers. The size of the aftermarket screw was different from that of the OEM screw; thus, the aftermarket screw exhibited a misfit with the OEM abutment. The FEA test indicated that the maximum von Mises stress of the aftermarket screw was 15.9% higher than that of the OEM screw under the same loading conditions. The system performance problems occurring with the aftermarket screw may not be visible to the naked eye in clinical use. Moreover, no immediate destructive failure would occur; thus, the aftermarket screw can still be used clinically. However, in some cases, such as trauma-related dental injuries or patients with parafunction habits (e.g., bruxism and clenching), the incisive force can be considerably higher than the physiological range^13,28^. If excessive force causes catastrophic damage to the implant–abutment–screw complex (such as screw fracture), physicians may need to remove or replace the complex surgically, which usually leads to extensive damage to the alveolar bone^13^. Therefore, in patients with the aforementioned risks, aftermarket screws should be cautiously used. We hope that manufacturers can enhance the accuracy of CAD/CAM for the clinical long-term success of implant prosthesis^9,13^.

In addition to the CAD/CAM error, the differences in the mechanical properties of the titanium alloys used in model II (aftermarket) and model I (OEM) may also cause problems in clinical use. These differences may cause complications in long-term implant use^13,29–31^. The microstructure and mechanical properties of the titanium alloys are affected by various manufacturing processes, such as thermomechanical and cold rolling processes^29^. The clinical performance of CAD/CAM titanium screws can be negatively affected if aftermarket manufacturers use raw materials that differ from those used by original manufacturers. Moreover, titanium machining is difficult, and that must be followed the machining guidelines include cutting at low speed, using sharp instruments, and never interrupting the cut procedure^30^. The mechanical performance of titanium screws can be significantly affected if aftermarket manufacturers fail to follow the machining guidelines. For example, poor processing can cause the discontinuity of the surface and reduce the ductility and fatigue properties of the metal. Surface overheating can result in the interstitial pickup of oxygen and nitrogen, which causes the machined titanium to be hard and brittle^31^.

In this study, the Zr abutment was used for FEA. Various materials, including titanium (Ti), Zr, and various metal alloys, are currently used for implant abutment fabrication. Due to their high strength and biocompatibility, Ti and metal alloy abutments are considered the standard choice for implant-supported restorations. However, Ti and metal abutments may show through the peri-implant soft tissue, resulting in greying of the marginal tissue and poor aesthetic outcomes. Hence, the Zr abutment was introduced in 1995 as a restorative alternative to metal abutments^14–16^. Zr has a modulus of elasticity of 200 GPa and flexural strength of 900 MPa. Moreover, the high fracture resistance of Zr improves the clinical success of aesthetic abutments^22,32,33^. Several studies have reported good survival rates and limited biological and technical challenges for Zr abutments^34–37^. Although Zr abutments offer several clinical advantages over metal abutments, clinicians still have doubts regarding the risk of abutment crack, especially when the screw material is different from the Zr abutment. The higher wear at the interface between titanium alloy and Zr^38,39^ as well as the internal stress concentration and friction properties for different materials should be examined.

In this study, the highest stress of the overall implant system was observed in the fixation screw for both the models. This finding is in agreement with those of previous FEA studies^17,40,41^. The differences in the maximum von Mises stress between model I and model II may have resulted from a misfit between the screw and the abutment. In this study, the maximum stress of the aftermarket screw was 15.9% higher than that of the OEM screw. Moreover, the maximum stress at the OEM abutment was 14.3% higher when using the aftermarket screw than when using the OEM screw. Screw misfit had a small effect on the maximum stresses of the implant and bone. Unsuitable connection between the screw and abutment may cause the compression of the implant screw, which results in torque loss, loosening of the screw, or fracture of the screw^13,42–45^. The highest stress was 526.97 MPa in model I and 611.52 MPa in model II. The aforementioned two stress values are lower than the yield strength of titanium alloy (760 MPa). This finding indicates that screw failure did not occur when a load of 170 N was applied obliquely on the implant system; however, model II, which had the highest stress in its narrowest part, was more susceptible to fatigue failure during clinical practice than model I was^17^.

The fracture point of the screw sometimes is located on the palatal side in the narrowest region (shank area) below the implant platform of the internal hex implant^4,7,8,40,46^. Moreover, with an external hex implant, screw fracture at the third thread region which presence of a lever around the screw^40^. In this study, with an internal hex implant, the maximum stress occurred on the palatal side of section B in model I and on the palatal side of section D in model II. Section B is the first step in the screw neck region, which lies in the suprabony area and is in contact with the corresponding abutment. However, clinical fracture does not occur easily at section B due to its large cross-sectional area. Removing broken screw fragments is easy due to their suprabony location. However, section D is the junction of the screw neck and shank. In the adopted screw configuration, section D is the narrowest region and is located in the infrabony area. Therefore, the maximum stress after loading was 15.9% higher for the aftermarket screw than it was for the OEM screw. Moreover, the position of maximum stress changed from section B (wide section) for the OEM screw to section D (narrowest section) for the aftermarket screw. In addition, the stress in sections D and E (narrowest sections) were 30.1% and 69.5% higher when using the aftermarket screw than when using the OEM screw. Therefore, we must be mindful of the irreversible complications that can occur during the clinical use of aftermarket CAD/CAM screws.

Problems in the abutment screw can cause serious complications. The screw is the smallest component in the implant system, and it connects the implant fixture with the abutment. In the study of Kreissl et al.^6^, the incidence of abutment screw fracture over a 5-year period was 3.9% and that of abutment screw loosening over the same period was 6.7%^6^. Although the screw loosening is not a critical complication in itself, it creates difficulties for both the dentist and patient. Screw loosening occurs when the compressive occlusal loadings are higher than the clamping force, which is the tension that holds the screw-abutment-implant together^47^. Another possible reason for screw loosening is screw defects. For example, the screw may be over-torqued when insertion or the misfit of the abutment when screw preload. The aforementioned defects result in increased stress on the screw, and unrecognised screw loosening may finally culminate in fracture of the screw^8^.

Few studies have examined aftermarket prosthetic components, and most of these studies have focused on aftermarket abutments with OEM screws. Gigandet et al.^48^ indicated that aftermarket CAD/CAM Zr abutments with OEM abutment screws may have low static load strength; thus, the suitability of these abutments for clinical use is questionable. Chang et al.^13^ investigated the mean maximum load capacity of OEM and aftermarket titanium abutments under static load and reported that OEM titanium abutments had significantly higher static load strength than the aftermarket abutments did. The failure mode involved deformation of the implant and abutment as well as fracture of the abutment and screw. Jarman et al.^9^ confirmed that the maximum load capacity of aftermarket Zr abutments was significantly lower than that of OEM Zr abutments. Kelly and Rungruanganunt^49^ reported that OEM abutments (Straumann) outperformed aftermarket abutments (Atlantis) under cyclic loading, and they concluded that the differences in abutment design and fabrication between aftermarket and OEM manufacturers can significantly influence the clinical performance of the abutments. The aforementioned results raise concerns regarding the use of aftermarket CAD/CAM prosthesis components. To the best of our knowledge, this study is the first to compare OEM and aftermarket titanium screws in an OEM zirconia abutment. The FEA results indicated that the maximum von Mises stress was higher in the aftermarket screws than it was in the OEM screws. This finding is in agreement with those of previous studies regarding aftermarket abutments^9,13,48,49^. Therefore, additional research and long-term clinical studies on the performance of aftermarket CAD/CAM screws are required to verify their reliability in clinical use.

## Conclusion

The following conclusions can be drawn from this study:

1. Significantly different measurements were obtained for the aftermarket CAD/CAM and OEM screws under 3D microscope. Therefore, the effect of the manufacturing differences between aftermarket and OEM screws on the clinical effect of aftermarket screws is unpredictable.
2. The FEA results indicated that under the same loading condition, the maximum stress of the aftermarket CAD/CAM screw was 15.9% higher than that of the OEM screw. Moreover, the maximum stress position occurred in a wide section of the OEM screw but the narrowest section of the aftermarket screw. The stress of the OEM Zr abutment was 14.9% higher when using the aftermarket screw than when using the OEM screw.
